# *catena*-Poly[[diaqua­zinc(II)]-μ-l-cystein­ato(2−)-κ^4^ *S*:*S*,*N*,*O*-[di-μ-sulfido-bis­[oxido­molybdate(V)](*Mo*—*Mo*)]-μ-l-cysteinato(2−)-κ^4^ *S*,*N*,*O*:*S*]

**DOI:** 10.1107/S1600536808007757

**Published:** 2008-04-02

**Authors:** Takashi Shibahara, Shinobu Ogasahara, Genta Sakane

**Affiliations:** a Department of Chemistry, Okayama University of Science, Ridai-cho, Okayama 700-0005, Japan

## Abstract

The title compound, [Mo_2_Zn(C_3_H_5_NO_2_S)_2_O_2_S_2_(H_2_O)_2_], forms a one-dimensional chain. The cysteine S atom of the dinuclear molybdenum complex anion coordinates to the zinc ion, which has a tetra­hedral environment by the additional coordination of two water mol­ecules. The one-dimensional chains are connected to each other by hydrogen bonds. The Zn—S(cysteine) distances [2.3599 (6) and 2.3072 (6) Å] are close to the value in ZnS (2.35 Å). The distances and angles within the complex are very close to those reported for the sodium and potassium di-μ-sulfide species.

## Related literature

For related literature, see: Brown & Jeffreys (1973 ▶); Hong *et al.* (1983 ▶); Kay & Mitchell (1970 ▶); Knox & Prout (1969 ▶); Shibahara *et al.* (1987 ▶); Lee *et al.* (1989 ▶); Liu & Williams (1981 ▶); Xing *et al.* (1998 ▶). 
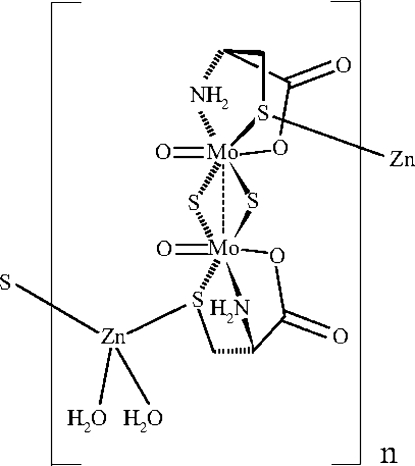


## Experimental

### 

#### Crystal data

[Mo_2_Zn(C_3_H_5_NO_2_S)_2_O_2_S_2_(H_2_O)_2_]
*M* *_r_* = 627.69
Monoclinic, 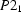

*a* = 8.6881 (11) Å
*b* = 10.3529 (8) Å
*c* = 9.8686 (11) Å
β = 108.2022 (14)°
*V* = 843.23 (16) Å^3^
*Z* = 2
Mo *K*α radiation
μ = 3.40 mm^−1^
*T* = 93.1 K
0.35 × 0.30 × 0.10 mm

#### Data collection

Rigaku Mercury diffractometer
Absorption correction: multi-scan (Jacobson, 1998 ▶) *T*_min_ = 0.382, *T*_max_ = 0.727
9357 measured reflections
4556 independent reflections
4549 reflections with *F* ^2^ > 2σ(*F* ^2^)
*R*_int_ = 0.019

#### Refinement

*R*[*F* ^2^ > 2σ(*F* ^2^)] = 0.019
*wR*(*F* ^2^) = 0.049
*S* = 1.02
4556 reflections
209 parameters
15 restraints
H atoms treated by a mixture of independent and constrained refinement
Δρ_max_ = 0.47 e Å^−3^
Δρ_min_ = −0.94 e Å^−3^
Absolute structure: Flack (1983 ▶), with 2010 Friedel pairs
Flack parameter: 0.002 (7)

### 

Data collection: *CrystalClear* (Rigaku, 1999 ▶); cell refinement: *CrystalClear*; data reduction: *CrystalStructure* (Rigaku, 2007 ▶); program(s) used to solve structure: *SIR97* (Altomare *et al.*, 1999 ▶); program(s) used to refine structure: *SHELXL97* (Sheldrick, 2008 ▶); molecular graphics: *CrystalStructure*; software used to prepare material for publication: *CrystalStructure*.

## Supplementary Material

Crystal structure: contains datablocks global, I. DOI: 10.1107/S1600536808007757/wk2080sup1.cif

Structure factors: contains datablocks I. DOI: 10.1107/S1600536808007757/wk2080Isup2.hkl

Additional supplementary materials:  crystallographic information; 3D view; checkCIF report

## Figures and Tables

**Table:** 

Mo1—S1	2.3201 (6)
Mo1—S2	2.3378 (6)
Mo1—S3	2.5572 (6)
Mo1—Mo2	2.8354 (3)
Mo2—S2	2.3276 (6)
Mo2—S1	2.3368 (6)
Mo2—S4	2.5428 (6)
Zn1—O8	2.0052 (17)
Zn1—O7	2.0275 (19)
Zn1—S4^i^	2.3072 (6)
Zn1—S3	2.3599 (6)

**Table:** 

O8—Zn1—O7	96.70 (7)
O8—Zn1—S4^i^	129.42 (5)
O7—Zn1—S4^i^	107.94 (6)
O8—Zn1—S3	93.73 (5)
O7—Zn1—S3	104.55 (6)
S4^i^—Zn1—S3	120.25 (2)

Symmetry code: (i) 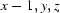.

**Table 2:** Hydrogen-bond geometry (Å, °)

*D*—H⋯*A*	*D*—H	H⋯*A*	*D*⋯*A*	*D*—H⋯*A*
O7—H11⋯O3^ii^	0.84	2.03	2.832 (2)	161
O8—H13⋯O5^ii^	0.84	1.77	2.604 (2)	171
O8—H14⋯O4^i^	0.84	2.00	2.789 (2)	158

Symmetry codes: (i) 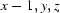; (ii) 
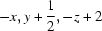
.

**Fig. 1.**
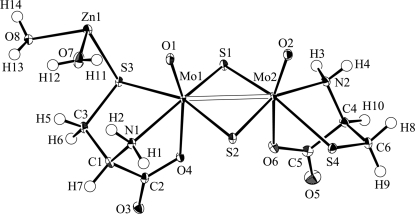
The asymmetric unit of I with atom labels and 50% probability displacement ellipsoids for non-H atoms.

**Fig. 2.**
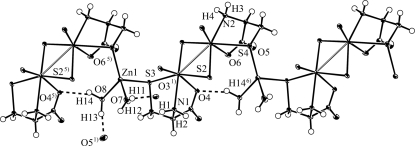
A view of part of a one-dimensional polymeric chain with hydrogen bonds (dashed lines).

## supplementary crystallographic information

### Comment

Molybdenum and *L*-cysteine are important components of many enzymes. X-ray structures of sulfur/oxygen-bridged dinuclear molybdenum complexes with *L*-cysteine ligands: Na_2_[Mo_2_(µ-S)_2_O_2_(cys)_2_].4H_2_O (Brown & Jeffreys, 1973; Hong *et al.*, 1983); K_2_[Mo_2_S_2_O_2_(cys)_2_].4H_2_O.CH_3_OH (Xing *et al.*, 1998), Ca[Mo_2_(µ-S)(µ-O)O_2_(cys)_2_].3H_2_O (Shibahara *et al.*, 1987), and Na_2_[Mo_2_O_4_(cys)_2_].5H_2_O (Knox & Prout, 1969; Kay & Mitchell, 1970; Liu & Williams, 1981), have been reported, where alkaline or alkaline earth metals are counter cations, and the existence of metal-oxygen (cysteine oxygen) bonds has been reported. Seeking another crystal structure type, we used Zn^2+^ion, as the counter ion. The present structural study of the complex compound Zn[Mo_2_O_2_S_2_(cys)_2_].2H_2_O (I) reveals the existence of Zn—S(cysteine sulfur) bonds, which result in polymerization; this type of Zn—S bond has been found in zinc finger proteins. The asymmetric unit of I is shown in Fig. 1 and a view of part of a one-dimensional polymeric chain of I is shown in Fig. 2. The zinc ion bridges the molybdenum complex anions: the coordination of the cysteine sulfur in the complex anion to the zinc ion results in the formation of one dimensional chains, where the zinc forms a tetrahedral structure by the additional coordination of two water molecules. The one dimensional chains are connected to each other by hydrogen bonds. Intra-chain hydrogen bonds also exist. The dimensions of the molybdenum complex and of the zinc tetrahedron are listed in Table 1, and the hydrogen bonds are listed in Table 2. The Zn—S(cysteine) distances (2.3599 (6), 2.3072 (6) Å) are close to that in ZnS (2.35 Å). The distances and angles within the complex are very close to those reported in the sodium and potassium salts in the di-*µ*-sulfide species.

### Experimental

The title compound was prepared by the addition of ZnCl_2_ to a diluted aqueous solution of Na_2_[Mo_2_O_2_S_2_(cys)_2_].4H_2_O. A crystal suitable for single-crystal X-ray diffraction was selected directly from the prepared sample.

### Refinement

H atoms bonded to C, N, and O (H_2_O) atoms were located in a difference map and refined with distance restraints of C—H = 0.99 (1), N—H = 0.92 (1), and O—H, 0.84 (1) Å, and with *U*_iso_(H) = 1.2*U*_eq_(C, N, O). The absolute structure was confirmed by the value of Flack parameter (0.003 (7)).

### Crystal data

**Table:**

[Mo_2_Zn(C_3_H_5_NO_2_S)_2_O_2_S_2_(H_2_O)_2_]	*F*_000_ = 612.00
*M**_r_* = 627.69	*D*_x_ = 2.472 Mg m^−^^3^
Monoclinic, *P*2_1_	Mo *K*α radiation λ = 0.71070 Å
Hall symbol: P 2yb	Cell parameters from 3004 reflections
*a* = 8.6881 (11) Å	θ = 5.5–30.0º
*b* = 10.3529 (8) Å	µ = 3.41 mm^−^^1^
*c* = 9.8686 (11) Å	*T* = 93.1 K
β = 108.2022 (14)º	Platelet, orange
*V* = 843.23 (16) Å^3^	0.35 × 0.30 × 0.10 mm
*Z* = 2	

### Data collection

**Table:**

Rigaku Mercury diffractometer	4549 reflections with *F*^2^ > 2σ(*F*^2^)
Detector resolution: 14.63 pixels mm^-1^	*R*_int_ = 0.019
ω scans	θ_max_ = 30.0º
Absorption correction: multi-scan(Jacobson, 1998)	*h* = −12→11
*T*_min_ = 0.382, *T*_max_ = 0.727	*k* = −14→14
9357 measured reflections	*l* = −13→13
4556 independent reflections	

### Refinement

**Table:**

Refinement on *F*^2^	*w* = 1/[σ^2^(*F*_o_^2^) + (0.0303*P*)^2^ + 0.5406*P*] where *P* = (*F*_o_^2^ + 2*F*_c_^2^)/3
*R*[*F*^2^ > 2σ(*F*^2^)] = 0.019	(Δ/σ)_max_ = 0.001
*wR*(*F*^2^) = 0.049	Δρ_max_ = 0.47 e Å^−^^3^
*S* = 1.02	Δρ_min_ = −0.94 e Å^−^^3^
4556 reflections	Extinction correction: none
209 parameters	Absolute structure: Flack (1983), with 2010 Friedel pairs
15 restraints	Flack parameter: 0.002 (7)
H atoms treated by a mixture of independent and constrained refinement	

### Special details

**Table:**

Geometry. ENTER SPECIAL DETAILS OF THE MOLECULAR GEOMETRY
Refinement. Refinement was performed using all reflections. The weighted R-factor (wR) and goodness of fit (S) are based on F^2^. R-factor (gt) are based on F. The threshold expression of F^2^ > 2.0 σ>F^2^) is used only for calculating R-factor (gt).

### Fractional atomic coordinates and isotropic or equivalent isotropic displacement parameters (Å^2^)

**Table:**

	*x*	*y*	*z*	*U*_iso_*/*U*_eq_	
Mo1	−0.056910 (19)	0.591229 (16)	0.808774 (16)	0.00615 (4)	
Mo2	0.113206 (19)	0.533750 (19)	0.613333 (16)	0.00647 (4)	
Zn1	−0.46728 (3)	0.59442 (3)	0.84366 (3)	0.01008 (4)	
S1	−0.07812 (7)	0.40574 (6)	0.67221 (6)	0.00917 (8)	
S2	0.16158 (6)	0.70395 (5)	0.77534 (6)	0.00880 (8)	
S3	−0.22984 (6)	0.46976 (5)	0.93439 (5)	0.00813 (8)	
S4	0.39318 (6)	0.59322 (6)	0.60271 (5)	0.01009 (8)	
O1	−0.2167 (2)	0.68209 (17)	0.71583 (17)	0.0104 (2)	
O2	0.00101 (19)	0.60344 (18)	0.45909 (16)	0.0111 (2)	
O3	0.2203 (2)	0.45050 (18)	1.21945 (17)	0.0125 (3)	
O4	0.13324 (19)	0.49461 (16)	0.98677 (16)	0.0089 (2)	
O5	0.4690 (2)	0.2383 (2)	0.7999 (2)	0.0217 (3)	
O6	0.2865 (2)	0.39663 (18)	0.77426 (17)	0.0125 (3)	
O7	−0.3954 (2)	0.77830 (18)	0.9006 (2)	0.0182 (3)	
O8	−0.54704 (19)	0.55572 (18)	1.00931 (17)	0.0136 (3)	
N1	−0.0163 (2)	0.70357 (19)	1.01408 (19)	0.0087 (3)	
N2	0.1744 (2)	0.3658 (2)	0.4962 (2)	0.0102 (3)	
C1	0.0084 (2)	0.6095 (2)	1.1320 (2)	0.0092 (3)	
C2	0.1322 (2)	0.5110 (2)	1.1161 (2)	0.0083 (3)	
C3	−0.1525 (2)	0.5421 (2)	1.1147 (2)	0.0099 (3)	
C4	0.3455 (2)	0.3284 (2)	0.5675 (2)	0.0112 (3)	
C5	0.3694 (2)	0.3165 (2)	0.7269 (2)	0.0116 (4)	
C6	0.4534 (2)	0.4378 (2)	0.5447 (2)	0.0130 (4)	
H1	0.0683	0.7596	1.0256	0.010*	
H2	−0.1027	0.7551	1.0124	0.010*	
H3	0.1090	0.2957	0.4919	0.012*	
H4	0.1624	0.3890	0.4039	0.012*	
H5	−0.2299	0.6042	1.1334	0.012*	
H6	−0.1366	0.4727	1.1869	0.012*	
H7	0.0460	0.6482	1.2291	0.011*	
H8	0.4318	0.4416	0.4403	0.016*	
H9	0.5706	0.4243	0.5928	0.016*	
H10	0.3821	0.2458	0.5359	0.013*	
H11	−0.3638	0.8290	0.8487	0.022*	
H12	−0.4303	0.8035	0.9659	0.022*	
H13	−0.5293	0.6197	1.0646	0.016*	
H14	−0.6424	0.5281	0.9816	0.016*	

### Atomic displacement parameters (Å^2^)

**Table:**

	*U*^11^	*U*^22^	*U*^33^	*U*^12^	*U*^13^	*U*^23^
Mo1	0.00760 (7)	0.00633 (8)	0.00567 (7)	0.00053 (6)	0.00374 (5)	0.00047 (6)
Mo2	0.00756 (7)	0.00729 (8)	0.00565 (7)	0.00067 (6)	0.00364 (5)	0.00022 (6)
Zn1	0.01030 (10)	0.01118 (12)	0.00928 (10)	0.00082 (10)	0.00382 (8)	−0.00105 (9)
S1	0.0114 (2)	0.0085 (2)	0.0091 (2)	−0.00148 (18)	0.00549 (17)	−0.00119 (17)
S2	0.0109 (2)	0.0077 (2)	0.0098 (2)	−0.00074 (18)	0.00601 (17)	−0.00107 (17)
S3	0.0087 (2)	0.0085 (2)	0.0086 (2)	−0.00003 (18)	0.00480 (16)	−0.00014 (17)
S4	0.0088 (2)	0.0127 (2)	0.0098 (2)	−0.0003 (2)	0.00436 (16)	0.00152 (19)
O1	0.0111 (6)	0.0117 (8)	0.0102 (6)	0.0024 (5)	0.0060 (5)	0.0015 (5)
O2	0.0113 (6)	0.0135 (8)	0.0097 (6)	0.0019 (6)	0.0048 (5)	0.0014 (6)
O3	0.0152 (7)	0.0123 (8)	0.0101 (6)	0.0030 (6)	0.0039 (5)	0.0014 (6)
O4	0.0102 (6)	0.0099 (7)	0.0074 (6)	0.0011 (5)	0.0041 (5)	0.0002 (5)
O5	0.0216 (8)	0.0185 (9)	0.0221 (8)	0.0064 (7)	0.0027 (7)	0.0083 (7)
O6	0.0166 (7)	0.0129 (8)	0.0087 (6)	0.0010 (6)	0.0049 (5)	−0.0004 (5)
O7	0.0217 (9)	0.0131 (8)	0.0250 (8)	−0.0031 (7)	0.0146 (7)	−0.0054 (7)
O8	0.0096 (6)	0.0217 (10)	0.0103 (6)	−0.0025 (5)	0.0045 (5)	−0.0045 (6)
N1	0.0111 (7)	0.0069 (8)	0.0096 (7)	0.0002 (6)	0.0053 (6)	0.0004 (6)
N2	0.0096 (7)	0.0125 (9)	0.0084 (7)	0.0010 (6)	0.0026 (6)	−0.0032 (6)
C1	0.0131 (8)	0.0086 (10)	0.0067 (7)	0.0002 (7)	0.0043 (6)	−0.0005 (7)
C2	0.0099 (8)	0.0060 (10)	0.0107 (8)	−0.0019 (6)	0.0058 (6)	−0.0008 (6)
C3	0.0118 (8)	0.0109 (9)	0.0088 (7)	−0.0005 (7)	0.0060 (6)	0.0001 (7)
C4	0.0114 (8)	0.0130 (10)	0.0088 (8)	0.0038 (7)	0.0024 (6)	−0.0032 (7)
C5	0.0126 (9)	0.0095 (10)	0.0124 (9)	−0.0007 (7)	0.0034 (7)	0.0003 (7)
C6	0.0117 (9)	0.0186 (11)	0.0099 (8)	0.0019 (7)	0.0052 (7)	−0.0024 (8)

### Geometric parameters (Å, °)

**Table:**

Mo1—O1	1.6905 (17)	O5—C5	1.238 (3)
Mo1—O4	2.2366 (16)	O6—C5	1.279 (3)
Mo1—N1	2.2662 (19)	O7—H11	0.837
Mo1—S1	2.3201 (6)	O7—H12	0.835
Mo1—S2	2.3378 (6)	O8—H13	0.841
Mo1—S3	2.5572 (6)	O8—H14	0.838
Mo1—Mo2	2.8354 (3)	N1—C1	1.481 (3)
Mo2—O2	1.6914 (16)	N1—H1	0.915
Mo2—N2	2.2419 (19)	N1—H2	0.917
Mo2—O6	2.3044 (18)	N2—C4	1.484 (3)
Mo2—S2	2.3276 (6)	N2—H3	0.914
Mo2—S1	2.3368 (6)	N2—H4	0.915
Mo2—S4	2.5428 (6)	C1—C3	1.523 (3)
Zn1—O8	2.0052 (17)	C1—C2	1.526 (3)
Zn1—O7	2.0275 (19)	C1—H7	0.995
Zn1—S4^i^	2.3072 (6)	C3—H5	0.989
Zn1—S3	2.3599 (6)	C3—H6	0.991
S3—C3	1.852 (2)	C4—C5	1.527 (3)
S4—C6	1.838 (3)	C4—C6	1.531 (4)
S4—Zn1^ii^	2.3072 (6)	C4—H10	0.996
O3—C2	1.237 (3)	C6—H8	0.989
O4—C2	1.290 (2)	C6—H9	0.989
			
O1—Mo1—O4	162.73 (7)	C6—S4—Zn1^ii^	102.50 (7)
O1—Mo1—N1	94.11 (7)	C6—S4—Mo2	99.79 (8)
O4—Mo1—N1	69.93 (6)	Zn1^ii^—S4—Mo2	99.22 (2)
O1—Mo1—S1	104.04 (6)	C2—O4—Mo1	119.10 (14)
O4—Mo1—S1	89.50 (4)	C5—O6—Mo2	117.99 (14)
N1—Mo1—S1	154.97 (5)	Zn1—O7—H11	123.4
O1—Mo1—S2	102.18 (6)	Zn1—O7—H12	110.6
O4—Mo1—S2	84.32 (4)	H11—O7—H12	122.9
N1—Mo1—S2	87.91 (5)	Zn1—O8—H13	109.3
S1—Mo1—S2	104.59 (2)	Zn1—O8—H14	111.1
O1—Mo1—S3	91.69 (6)	H13—O8—H14	116.3
O4—Mo1—S3	78.52 (4)	C1—N1—Mo1	107.99 (13)
N1—Mo1—S3	76.70 (5)	C1—N1—H1	113.9
S1—Mo1—S3	85.55 (2)	Mo1—N1—H1	109.3
S2—Mo1—S3	160.022 (19)	C1—N1—H2	108.3
O1—Mo1—Mo2	105.18 (6)	Mo1—N1—H2	112.6
O4—Mo1—Mo2	91.51 (4)	H1—N1—H2	104.8
N1—Mo1—Mo2	138.35 (5)	C4—N2—Mo2	108.76 (13)
S1—Mo1—Mo2	52.760 (15)	C4—N2—H3	109.3
S2—Mo1—Mo2	52.411 (14)	Mo2—N2—H3	113.3
S3—Mo1—Mo2	137.441 (15)	C4—N2—H4	109.0
O2—Mo2—N2	91.69 (8)	Mo2—N2—H4	109.8
O2—Mo2—O6	162.14 (7)	H3—N2—H4	106.6
N2—Mo2—O6	70.64 (6)	N1—C1—C3	108.11 (17)
O2—Mo2—S2	103.00 (6)	N1—C1—C2	107.01 (16)
N2—Mo2—S2	156.97 (5)	C3—C1—C2	109.49 (18)
O6—Mo2—S2	93.10 (5)	N1—C1—H7	114.5
O2—Mo2—S1	102.18 (6)	C3—C1—H7	107.9
N2—Mo2—S1	89.44 (5)	C2—C1—H7	109.8
O6—Mo2—S1	80.89 (5)	O3—C2—O4	123.7 (2)
S2—Mo2—S1	104.38 (2)	O3—C2—C1	121.88 (18)
O2—Mo2—S4	98.59 (6)	O4—C2—C1	114.38 (18)
N2—Mo2—S4	77.44 (5)	C1—C3—S3	109.75 (13)
O6—Mo2—S4	75.53 (5)	C1—C3—H5	109.6
S2—Mo2—S4	82.83 (2)	S3—C3—H5	112.2
S1—Mo2—S4	155.71 (2)	C1—C3—H6	109.6
O2—Mo2—Mo1	104.26 (5)	S3—C3—H6	109.0
N2—Mo2—Mo1	140.49 (5)	H5—C3—H6	106.7
O6—Mo2—Mo1	91.59 (4)	N2—C4—C5	107.66 (18)
S2—Mo2—Mo1	52.737 (15)	N2—C4—C6	107.82 (19)
S1—Mo2—Mo1	52.226 (15)	C5—C4—C6	108.60 (18)
S4—Mo2—Mo1	133.300 (15)	N2—C4—H10	116.5
O8—Zn1—O7	96.70 (7)	C5—C4—H10	107.4
O8—Zn1—S4^i^	129.42 (5)	C6—C4—H10	108.7
O7—Zn1—S4^i^	107.94 (6)	O5—C5—O6	125.6 (2)
O8—Zn1—S3	93.73 (5)	O5—C5—C4	119.9 (2)
O7—Zn1—S3	104.55 (6)	O6—C5—C4	114.4 (2)
S4^i^—Zn1—S3	120.25 (2)	C4—C6—S4	110.80 (15)
Mo1—S1—Mo2	75.014 (19)	C4—C6—H8	104.8
Mo2—S2—Mo1	74.853 (18)	S4—C6—H8	108.1
C3—S3—Zn1	98.94 (7)	C4—C6—H9	114.2
C3—S3—Mo1	100.08 (7)	S4—C6—H9	109.0
Zn1—S3—Mo1	97.09 (2)	H8—C6—H9	109.7

Symmetry codes: (i) *x*−1, *y*, *z*; (ii) *x*+1, *y*, *z*.

### Hydrogen-bond geometry (Å, °)

**Table:**

*D*—H···*A*	*D*—H	H···*A*	*D*···*A*	*D*—H···*A*
O7—H11···O3^iii^	0.84	2.03	2.832 (2)	161
O7—H11···O4^iii^	0.84	2.75	3.139 (2)	110
O7—H12···O5^iii^	0.84	2.53	3.239 (3)	144
O7—H12···O6^iii^	0.84	2.66	3.285 (2)	133
O7—H12···O8^iv^	0.84	2.64	3.093 (2)	116
O8—H13···O5^iii^	0.84	1.77	2.604 (2)	171
O8—H14···O4^i^	0.84	2.00	2.789 (2)	158
O8—H14···O6^i^	0.84	2.37	2.844 (2)	116
N1—H2···O4^iii^	0.92	2.49	3.179 (2)	132
N1—H2···O7	0.92	2.45	3.224 (2)	143
N2—H3···O2^v^	0.91	2.32	3.212 (2)	164
N2—H4···O1^v^	0.92	2.56	2.934 (2)	105
N2—H4···O3^vi^	0.92	2.13	3.011 (2)	161

Symmetry codes: (iii) −*x*, *y*+1/2, −*z*+2; (iv) −*x*−1, *y*+1/2, −*z*+2; (i) *x*−1, *y*, *z*; (v) −*x*, *y*−1/2, −*z*+1; (vi) *x*, *y*, *z*−1.
